# First-Attempt Intubation Success Rate Using the C-MAC Videolaryngoscope Versus Direct Laryngoscopy Among Anaesthesiology Residents Performing Rapid Sequence Induction: A Randomised Controlled Trial

**DOI:** 10.7759/cureus.102723

**Published:** 2026-01-31

**Authors:** Penumaka Lava Kumar, Sakthirajan Panneerselvam, Santhosh Arulprakasam, Priya Rudingwa, Ranjithkumar Mekala, Prasanna U Bidkar

**Affiliations:** 1 Anaesthesiology, All India Institute of Medical Sciences (AIIMS), Mangalagiri, IND; 2 Anaesthesiology and Critical Care, Jawaharlal Institute of Postgraduate Medical Education and Research, Puducherry, IND

**Keywords:** academic training, airway management, endotracheal intubation, hypoxia, laryngoscopes

## Abstract

Background and aims: The primary objective of this randomised trial was to compare the first-attempt intubation success rate between the C-MAC videolaryngoscope (VL) and the Macintosh direct laryngoscope (DL) when used by novice anaesthesia residents during rapid sequence induction (RSI). Secondary objectives were to compare intubation time; laryngoscopic view (Cormack-Lehane grade (CLG) and Percentage of Glottic Opening (POGO) score); the need for adjunct manoeuvres and devices (optimal external laryngeal manipulation (OELM), release of cricoid pressure, and use of a gum elastic bougie (GEB)); and the incidence of hypoxia, regurgitation, mucosal or dental injury, and haemodynamic changes during and after intubation.

Methods: This randomised controlled trial included 172 adult patients with normal airways requiring RSI, who were allocated to either the VL group (n = 86) or the DL group (n = 86). Resident anaesthesiologists underwent lecture- and simulation-based training prior to performing intubations in the operating room. RSI and tracheal intubation were performed using the allocated device. The primary endpoint was first-attempt intubation success. Secondary endpoints included intubation time, manoeuvres required for successful intubation, incidence of hypoxia and regurgitation, mucosal or dental injury, and haemodynamic changes during and after intubation.

Results: First-attempt intubation success was significantly higher in the VL group compared with the DL group (93% vs 67%, P < 0.001). A CLG 1 view was obtained in 76% of patients in the VL group and 54% in the DL group (P < 0.001). External laryngeal manipulation, release of cricoid pressure, use of a GEB, and dental injuries were significantly lower in the VL group (P < 0.001). Heart rate and systolic blood pressure increased significantly during DL-guided intubation.

Conclusion: VL-guided intubation by novice anaesthesia residents during RSI was associated with a higher first-attempt success rate and superior laryngoscopic views compared with DL, along with fewer adjunct manoeuvres and airway injuries. These findings support the use of VL as a valuable tool for resident-performed RSI in patients without anticipated difficult airways.

## Introduction

Videolaryngoscopes (VLs) have gained popularity over direct laryngoscopes (DLs) because they provide an improved laryngeal view, facilitating tracheal intubation, particularly in difficult airway situations. Multiple studies and meta-analyses have demonstrated that VLs increase first-attempt intubation success rates compared with DLs in emergency and critical care settings, especially when used by less experienced operators such as trainees or novices [1-3]. A retrospective study of emergency department intubations reported significantly higher first-pass success rates with the C-MAC VL compared with DL, even when emergency personnel were wearing personal protective equipment. Similarly, meta-analyses have confirmed the advantages of VL in improving first-pass success and reducing airway trauma during intubation in neonates and critically ill adults [4-7].

Rapid sequence induction (RSI) is commonly used in emergency airway management to minimise the risk of aspiration. However, RSI presents unique challenges, including potential distortion of the laryngeal view due to cricoid pressure and exaggerated haemodynamic responses during intubation. Conventional DL using Macintosh blades remains standard practice, yet repeated or failed intubation attempts during RSI increase the risk of complications, such as hypoxemia and airway trauma. The C-MAC VL (Karl Storz SE & Co. KG, Tuttlingen, Germany) combines a familiar Macintosh-style blade with video-guided visualisation, potentially allowing operators to benefit from improved laryngeal views without major changes in technique or extensive additional training [8-11].

Although first-pass success rates with VLs and DLs are comparable among experienced clinicians, VLs have demonstrated superior success rates when used by trainees or less experienced clinicians during emergency intubations in intensive care and emergency settings. Additionally, the use of VL during emergency RSI may reduce the need for adjunct manoeuvres, decrease mucosal injury, and attenuate haemodynamic fluctuations associated with intubation-related stress [12].

The primary objective of this study was to compare the first-attempt intubation success rate between the C-MAC VL and the Macintosh DL during RSI performed by novice anaesthesiology residents. Secondary objectives included comparisons of laryngoscopic view (Cormack-Lehane grade (CLG) and Percentage of Glottic Opening (POGO) score); time to successful intubation; the need for adjunct manoeuvres and devices (optimal external laryngeal manipulation (OELM), release of cricoid pressure, and gum elastic bougie (GEB)); incidence of hypoxia, regurgitation, and mucosal and dental injury; and haemodynamic changes during and after intubation between the two groups.

## Materials and methods

Study design and participants

This randomised controlled trial was conducted from September 2021 to February 2023 after obtaining approval from the Institutional Ethics Committee (JIP/IEC/2021/042, dated 16 April 2021) and registration with the Clinical Trials Registry of India (CTRI/2021/08/035986). Adult patients aged 18-60 years, with American Society of Anaesthesiologists Physical Status (ASA-PS) I-III, and requiring RSI for emergency or elective surgical procedures under general anaesthesia were included. Patients with an anticipated difficult airway, obesity, obstructive sleep apnoea (OSA), cervical spine injury, preoperative oxygen therapy, vasopressor treatment, or parturients were excluded. An anticipated difficult airway was defined by the presence of one or more of the following on preoperative assessment: Mallampati class III-IV, inter-incisor distance < 3 cm, thyromental distance < 6 cm, restricted neck movement, or a documented history of difficult intubation. Written informed consent was obtained from all enrolled patients. The study was conducted in accordance with the principles of the Declaration of Helsinki (1964) and its subsequent amendments, and adhered to the Consolidated Standards of Reporting Trials (CONSORT) guidelines [13]. 

Laryngoscopy and intubation were performed by anaesthesia residents with 12-18 months of training. Residents underwent lecture-based instruction and simulation training on manikins until they achieved three consecutive successful intubations within 60 seconds using each technique. Experienced anaesthesiologists demonstrated both techniques on anaesthetised patients, after which residents performed VL-guided intubations under supervision. A trained anaesthesiologist not involved in the study performed and documented the preoperative airway assessment. All patients received aspiration prophylaxis according to institutional protocol.

After obtaining informed consent, patients were transferred to the operating room. Standard ASA monitors were applied, and baseline parameters were recorded. Appropriate rescue airway equipment, including face masks, oropharyngeal and nasopharyngeal airways, laryngeal mask airways, GEBs, and stylets, was prepared. Patients were positioned in the sniffing position. Pre-oxygenation was performed using a closed circuit with 100% oxygen at a flow rate of 8-10 L/min for at least three minutes or until an end-tidal oxygen concentration of 90% was achieved. Randomisation was performed by an independent individual using computer-generated block randomisation with variable block sizes of 4-8. Allocation concealment was ensured using sealed opaque envelopes, which were opened by a senior anaesthesiologist during pre-oxygenation. Based on the group assignment, residents used either the DL or VL to visualise the glottis and perform tracheal intubation. In both groups, blade size was selected based on externally measured mandibular depth. A malleable stylet lubricated with 2% lidocaine jelly was inserted into the endotracheal tube (ETT) to a safe length and shaped into a hockey-stick configuration to match the curvature of the respective laryngoscope blade.

Anaesthesia was induced with propofol 2 mg kg⁻¹, followed by either succinylcholine 2 mg kg⁻¹ or rocuronium 1.2 mg kg⁻¹, with concurrent application of cricoid pressure after informing the patient. The resident maintained an adequate mask seal to provide continuous oxygenation during the apnoeic period. Laryngoscopy and intubation were attempted 60 seconds after administration of the neuromuscular blocking agent using the allocated device. OELM was applied at the request of the laryngoscopist to improve glottic exposure. Cricoid pressure was released or adjusted, if necessary, after confirmation with the supervising anaesthesiologist. If required, a GEB was provided after removal of the stylet to facilitate intubation. Haemodynamic parameters were recorded at one-minute intervals for 10 minutes during and after laryngoscopy and intubation. Following successful intubation, the ETT was secured at an appropriate depth based on bilateral chest rise and auscultation of equal breath sounds, and connected to the ventilator. Airway pressures and minute ventilation were monitored to ensure adequate ventilation. If first-attempt intubation was unsuccessful, subsequent attempts were performed by a senior anaesthesiologist using a laryngoscope of their choice.

Primary outcome

The primary outcome was the first-attempt intubation success rate, defined as successful placement of the ETT through the glottis with attainment of a CLG and POGO score, with or without optimisation manoeuvres, and confirmation by continuous waveform capnography. The total intubation time was defined as the interval from insertion of the laryngoscope blade beyond the incisors to the appearance of a sustained four-square waveform of end-tidal carbon dioxide (EtCO₂).

Secondary outcome

The secondary outcomes included the incidence of hypoxia (defined as SpO₂ < 90%) and regurgitation, the occurrence of mucosal or dental injury, and haemodynamic changes during and after intubation. Haemodynamic changes were defined as an increase in systolic blood pressure (SBP) of >20% from baseline, with a post-intubation SBP exceeding 160 mmHg.

Statistical analysis

Continuous variables, such as age, weight (kg), and BMI (kg m⁻²), were summarized as mean ± standard deviation (SD). Total intubation time was analysed using the Mann-Whitney U test due to non-normal distribution but is presented as mean ± SD for consistency. Categorical variables, including gender, were expressed as frequency and proportion. CLG, POGO score, the need for intubation aids, adjustment or release of cricoid pressure, dental injury, and mucosal injury were expressed as relative risk (RR) with 95% confidence intervals (CI) and analysed using Fisher’s exact test. The requirement for OELM was expressed as RR (95% CI) and analysed with the chi-square test. Baseline heart rate (HR) and SBP, as well as maximum HR and SBP during laryngoscopy and for the 10 minutes following intubation, were expressed as mean ± SD and analysed using a mixed-effects linear model with restricted maximum likelihood (REML) estimation. A two-sided P-value < 0.05 was considered statistically significant. All analyses were performed using STATA version 14.0 (StataCorp LLC, College Station, TX, USA).

As multiple intubations were performed by the same residents, there is a potential for clustering by operator. In the present analysis, each intubation was treated as an independent observation without adjustment for clustering. This approach may have led to slightly narrower confidence intervals, and this limitation is acknowledged in the Discussion section.

Sample size estimation

Based on Baek et al. [14], the first-pass intubation success rate was 79.3% with a VL and 59.7% with a DL, representing a 20.6% higher success rate with VL. Assuming a power of 80%, a 95% CI, and accounting for 10% attrition, a total of 172 participants were recruited. Sample size calculations were performed using nMaster version 2.0 (Department of Biostatistics, Christian Medical College, Vellore, India).

## Results

**Figure 1:**
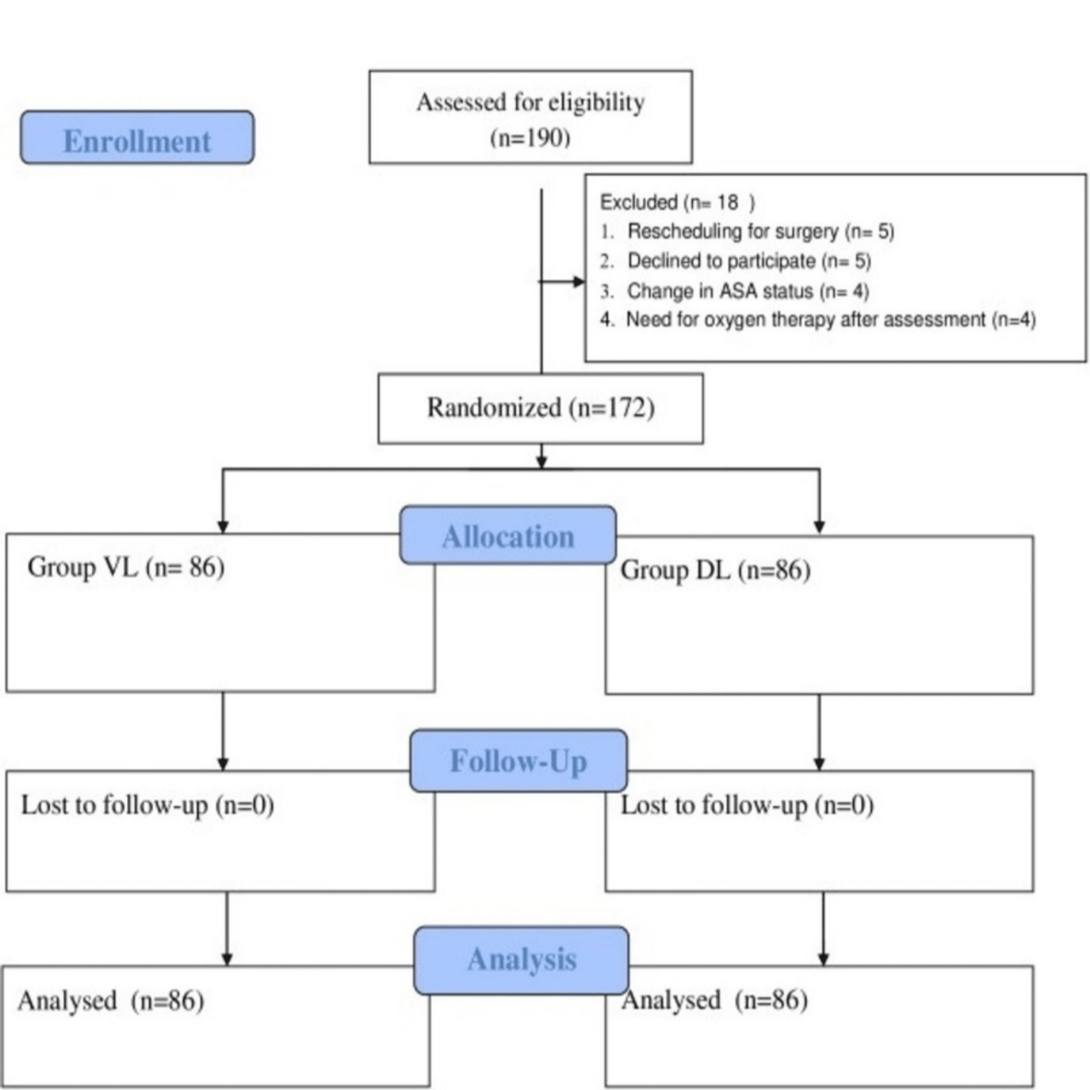
CONSORT diagram of the patient selection process

A total of 190 patients were assessed for eligibility. Eighteen patients were excluded due to rescheduling of surgery (n = 5), declining participation (n = 5), change in ASA status (n = 4), or requirement for oxygen therapy after assessment (n = 4). The remaining 172 patients were randomised, with 86 assigned to each group (Figure 1).

Table 1 summarises the characteristics of the study participants.

**Table 1:** Demographic characteristics of the study participants Data are presented as mean ± standard deviation and frequency.

Variable	Videolaryngoscope (n = 86)	Direct laryngoscope (n = 86)
Age (years)	43 ± 12.2	43.5 ± 12
Male	45 (52%)	54 (62%)
Female	41 (48%)	32 (38%)
Weight (kg)	63 ± 9.5	62 ± 9.6
BMI (kg/m^2^)	23.4 ± 3.1	23 ± 3.1

In the VL group, 80 patients (93.0%) were successfully intubated on the first attempt, compared with 58 patients (67.4%) in the DL group. The VL group also demonstrated superior laryngoscopic views, with better CLG and higher POGO scores compared with the DL group (Tables 2, 3).

**Table 2:** Parameters of laryngoscopic views (Cormack-Lehane grade and POGO score) Data are expressed as number (percentages). POGO: Percentage of Glottic Opening. *Fisher’s exact test.

Outcome		Videolaryngoscope (n = 86)	Direct laryngoscope (n = 86)	RR (95% CI)	P-value^*^	P-value*
Cormack-Lehane grade	1	66 (76.7%)	47(54.6%)	1.4 (1.12-1.76)	0.002	0.001
	2A	15 (17.4%)	16 (18.6%)	0.73 (0.39-1.35)	0.32	
	2B	5 (5.8%)	19 (22.0%)	0.25 (0.09-0.61)	0.001	
	3	0 (0%)	4 (4.6%)	-	0.03	
POGO score	100	65 (75.5%)	48 (42.4%)	1.35 (1.08-1.69)	0.006	0.006
	75	15 (17.4%)	17 (19.7%)	0.71 (0.38-1.32)	0.29	
	50	6 (6.9%)	18 (20.9%)	0.31 (0.13-0.73)	0.004	
	25	0 (0%)	3 (3.4%)	-	0.08	

**Table 3:** Intubation success and manoeuvres needed for successful intubation Data are expressed as number (percentages) or mean ± SD. GEB: gum elastic bougie, OELM: optimal external laryngeal manipulation. *Fisher's exact test. ^†^Chi-square test. ^‡^Mann-Whitney U test.

Outcome	Videolaryngoscope (n = 86)	Direct laryngoscope (n = 86)	RR (95% CI)	P-value
					Primary outcome				
First-attempt success	80 (93.02%)	58 (67.44%)	1.37 (1.17-1.61)	<0.001^*^
Categorical secondary outcome				
GEB-guided intubation	4 (4.6%)	54 (62.7%)	0.07 (0.03-0.2)	<0.001^*^
Cricoid pressure released	4 (4.6%)	19 (22%)	0.21 (0.07-0.59)	<0.001^*^
Requirement of OELM to optimise the laryngeal view	6 (6.9%)	43 (50%)	0.14 (0.06-0.31)	<0.001^†^
Continuous secondary outcome				
Time to successful intubation (seconds)	39.3 ± 1.3	43.9 ± 1.9	41.6 (39.3-43.9)	0.39^‡^

The VL group achieved successful intubation in a shorter time compared with the DL group, although the difference was not statistically significant (39.3 vs 43.9 seconds, P = 0.39). The DL group had higher incidences of GEB use, cricoid pressure release, and requirement for OELM compared with the VL group (P = 0.001) (Table 3). No episodes of hypoxia, regurgitation, or aspiration were observed in either group. Mucosal injuries occurred in 5.8% of patients (n = 5) in the DL group, while none were reported in the VL group (P = 0.05). Dental injuries were observed in 2.4% of patients (n = 2) in the DL group, with none in the VL group (P = 0.4).

The DL group exhibited a higher maximum HR during laryngoscopy and in the 10 minutes following intubation compared with the VL group (β-estimate = -7.8, 95% CI: -9.8 to -5.7, P < 0.001). Maximum SBP during laryngoscopy and the subsequent 10 minutes was also higher in the DL group, though the difference was not statistically significant (β-estimate = 1.9, 95% CI: -0.67 to 4.5, P = 0.15). Haemodynamic parameters were analysed using a linear mixed-effects model (Table 4).

**Table 4:** Haemodynamic parameters Data are expressed as mean ± SD and OR (95% CI), with β-estimate. VL: videolaryngoscope, DL: direct laryngoscope, HR: heart rate, SBP: systolic blood pressure. *Mixed-effects linear model; restricted maximum likelihood (RML).

Variables		VL group (n = 86)	DL group (n = 86)	Change in HR/SBP from baseline to 10 minutes after intubation		P-value*
				β -estimate	95% CI	
Heart rate (bpm)	Baseline	72.95 ± 10.62	82.47 ± 15.81	-7.8	-5.7 to -9.8	<0.001
	Maximum HR during laryngoscopy	90.55 ± 9.48	97.58 ± 13.12			
	Maximum HR during the next 10 minutes after intubation	93.10 ± 8.82	99.98 ± 13.12			
SBP (mm Hg)	Baseline	125.91± 10.48	125.84 ± 17.23	1.9	-0.67 to 4.5	0.15
	Maximum SBP during laryngoscopy	147.16 ±10.53	145.81 ± 20.99			
	Maximum SBP during the next 10 minutes after intubation	148.75±11.61	144.39±16.28			

## Discussion

This trial demonstrated that VL-guided intubation during rapid sequence induction was successful on the first attempt in 93% of patients, compared with 67% with DL-guided intubation by novice anaesthesia residents. Among experienced clinicians, first-pass success rates for DL and VL are similar, with 100% success attributed to operator expertise [2]. Sakles et al. reported that novice emergency medicine residents achieved first-attempt success rates of 87.6% with C-MAC VL versus 75.3% with Macintosh DL [15]. Similarly, first-year emergency residents performing cardiopulmonary resuscitation achieved 91% success with the GlideScope compared with 55% with the DL after eight hours of VL training [8]. A meta-analysis in obese patients also demonstrated higher first-pass success with VL compared to DL (P = 0.0064) [16], consistent with our findings.

The higher success with VL is likely attributable to better glottic visualisation and a shorter learning curve, as the C-MAC shares a similar blade design and technique with conventional DL, facilitating quicker familiarisation. Previous studies have demonstrated high success rates of VL-guided intubation in simulation, emergency, intensive care, and out-of-hospital settings [4,9,17,18]. In contrast, the lower DL success rate may reflect residents’ limited experience and restricted glottic visualisation.

Rapid sequence intubation in the operating room can be challenging due to time pressure and workload, which may affect intubation success and distort the laryngeal view because of applied cricoid pressure. Current difficult airway guidelines suggest releasing cricoid pressure if intubation is difficult; however, this may increase the risk of regurgitation and aspiration [19]. In our study, the need to release cricoid pressure was significantly lower with C-MAC VL-guided intubation, likely due to improved Cormack-Lehane glottic visualisation despite cricoid pressure application.

VL may theoretically reduce aspiration risk by minimising the need for cricoid pressure release compared with DL; however, no regurgitation or aspiration events occurred in this study, so this potential benefit remains speculative and warrants confirmation in larger trials. Although guidelines vary, current evidence supports performing cricoid pressure based on clinician judgment and patient-specific factors [20]. 

In our study, intubation time was similar between DL- and VL-guided intubation. This contrasts with other studies reporting shorter intubation times with VL [5,8,9]. A separate analysis could have clarified how glottic visualisation or other factors contributed to delays. The frequent use of a GEB in the DL group may explain why intubation time remained similar despite direct visualisation. A meta-analysis of emergency intubations outside the operating room found that, although VL improved first-pass success, it did not reduce intubation time. While VL use by novice clinicians has been associated with post-intubation hypotension [21], maximum post-intubation blood pressure was comparable between groups in our study, with no episodes of hypotension. A recent Cochrane review of 222 RCTs indicates that VL, regardless of design, is more likely than DL to reduce complications such as hypoxemia, mucosal or dental trauma, and oesophageal intubation, while improving first-pass success and glottic visualisation [22]. 

In our study, 62% of GEB-assisted intubations occurred in the DL group. This frequent use likely reflects lower CLG and reduced glottic visualisation with DL, prompting residents to prefer bougies over stylets. Interestingly, in ICU emergency intubations by non-expert physicians, no difference in bougie use between DL and VL was observed, which the authors attributed to regional practices in Europe where stylets are less commonly used [3]. Cricoid pressure further impaired glottic views during RSI, and GEB-assisted intubation has been shown to improve first-pass success and reduce intubation time compared with stylets [23]. Despite 54% of patients achieving CLG 1, the increased bougie use in our cohort may reflect residents’ relative unfamiliarity with stylet-guided intubation using a DL.

The C-MAC VL outperformed DL, demonstrating higher first-attempt success (80%-85% vs. 65%-70%), superior CLG 1/2 glottic views (>90% vs. 80%-85%), reduced need for adjuncts, fewer airway injuries, better haemodynamic stability, and a shorter learning curve for novice operators in challenging RSI scenarios.

This study has several limitations. First, supervising anaesthesiologists could view the glottic image on the VL screen and provide guidance, potentially introducing supervision-related bias, which is less likely with DL. Residents also had unequal prior experience with the two devices, and multiple intubations were performed by the same residents, raising the possibility of operator clustering affecting outcomes. The protocol combined the time to glottic view and time to intubation into a single endpoint, which may have obscured differences between these components. Additionally, no episodes of hypoxia, regurgitation, or aspiration occurred, limiting conclusions about safety. High-risk or anticipated difficult airways were excluded, restricting the applicability of findings to these populations. Finally, as a single-centre study conducted in a supervised operating room, results may not generalise to unsupervised, emergency, or pre-hospital settings.

## Conclusions

VL-guided intubation demonstrated superior performance compared with conventional DL during rapid sequence induction by novice anaesthesia residents. In our study, VL was associated with higher first-attempt success, improved laryngoscopic views, reduced need for external laryngeal manoeuvres and airway adjuncts, and more stable haemodynamic responses. These results support the use of VL as a valuable tool for resident-performed RSI in patients without anticipated difficult airways. Further research is warranted to confirm its advantages in complex airway scenarios and in non-operating room settings.

## Figures and Tables

**Figure 2:**
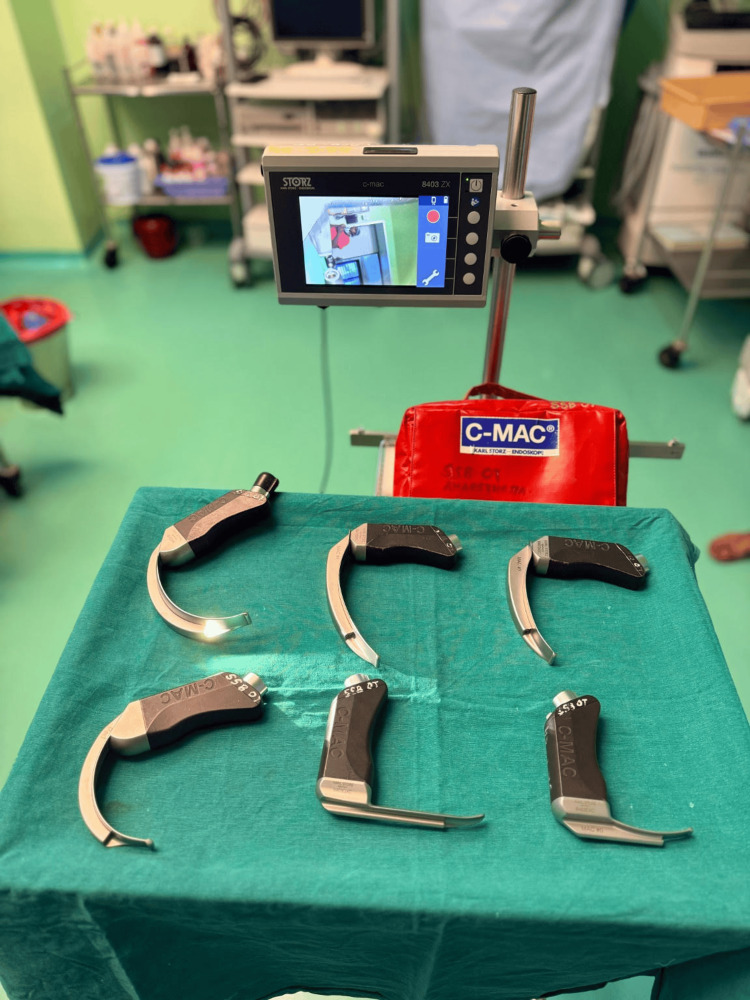
Karl Storz C-MAC® videolaryngoscopy system (8403 ZX monitor) with multiple reusable C-MAC blades arranged on the operating table

**Figure 3:**
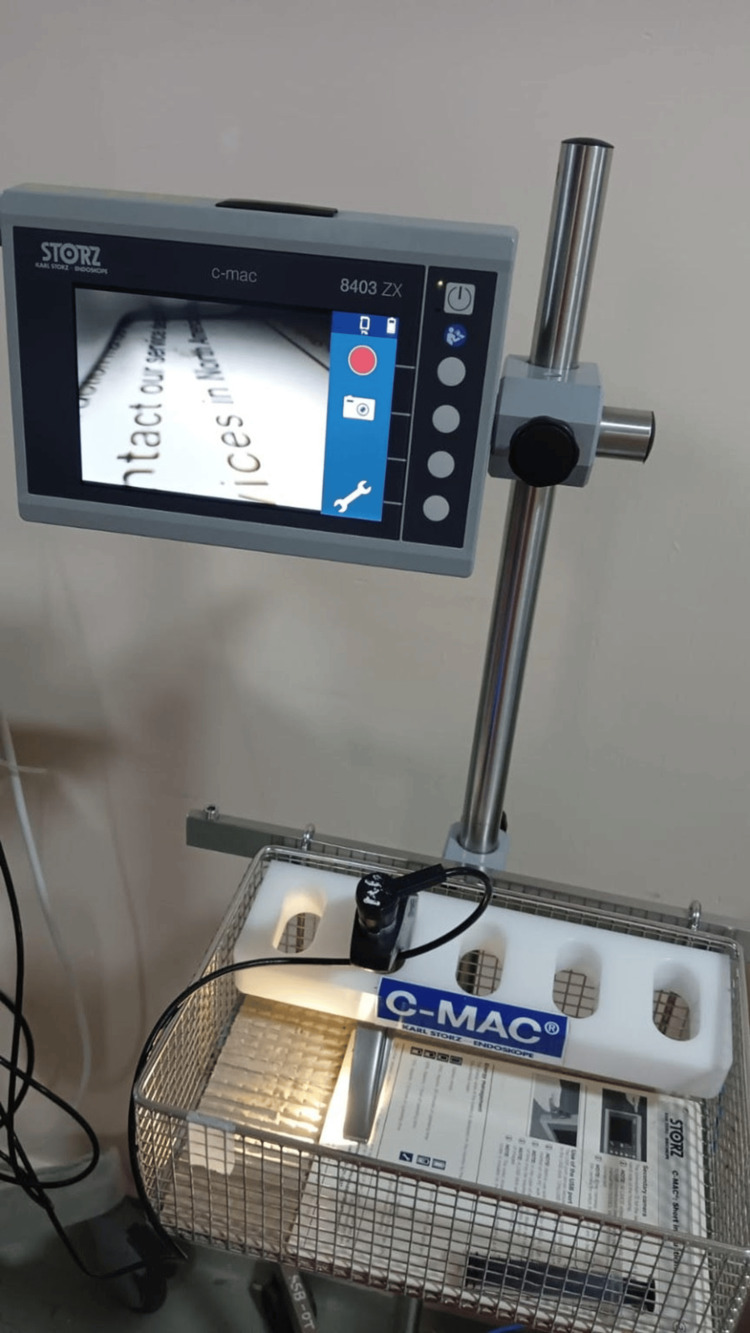
Karl Storz C-MAC® videolaryngoscope monitor (Model 8403 ZX) mounted on a stand

**Figure 4:**
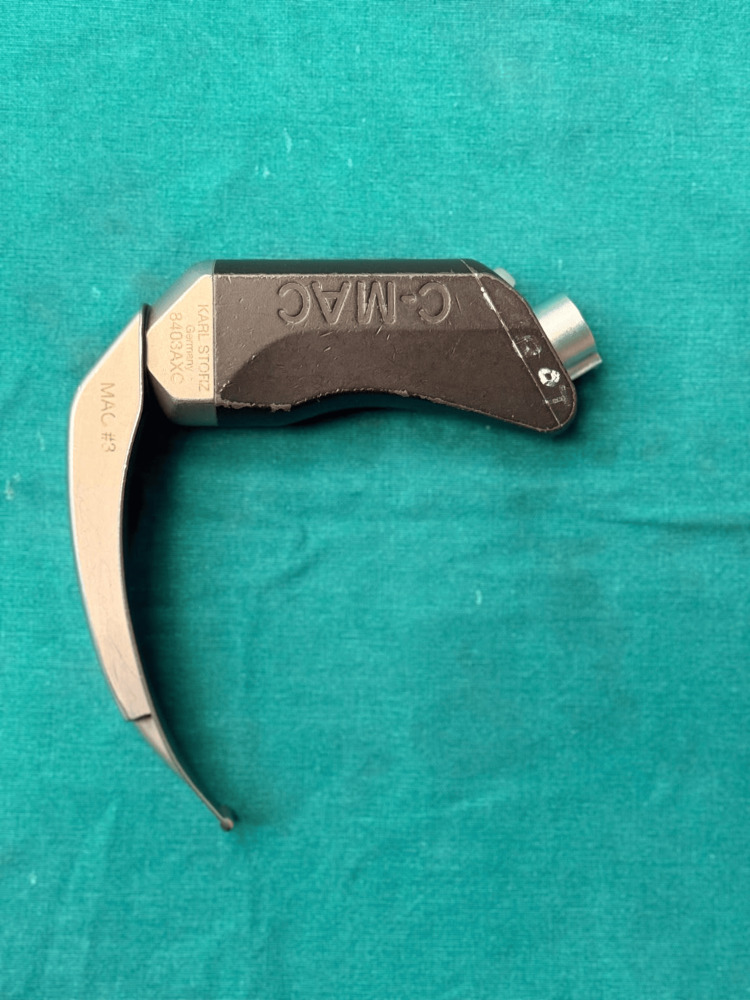
C-MAC® Macintosh blade size 3 (MAC 3) Reusable video laryngoscope blade with integrated camera and light source

**Figure 5:**
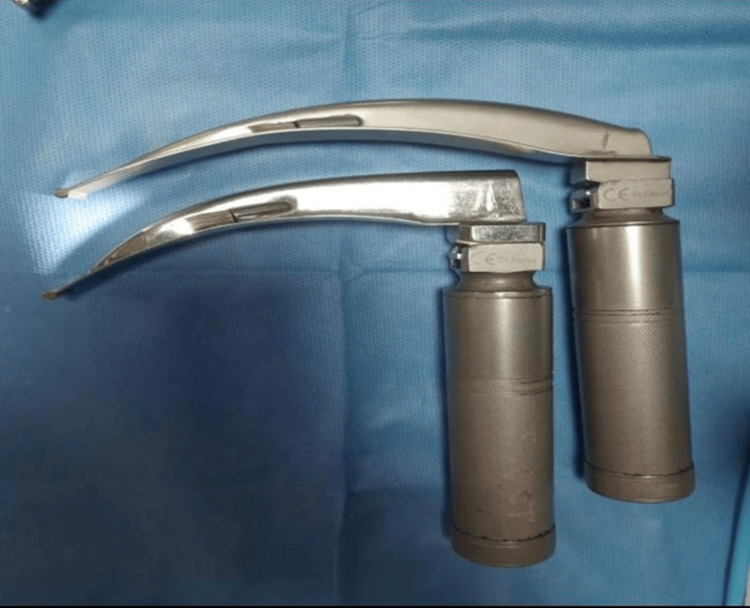
Conventional direct laryngoscope Macintosh blades with handles

## Appendices

The following photographs show the Karl Storz C-MAC® video laryngoscopy system, including the monitor and different blade types. Each image has been appropriately labeled for ease of reference. These are original photographs taken in our operating theatre/equipment area and are provided solely for clarification and documentation purposes. No patient identifiers are included in any of the images.

## References

[1] Kim JW, Park SO, Lee KR (2016). Video laryngoscopy vs. direct laryngoscopy: which should be chosen for endotracheal intubation during cardiopulmonary resuscitation? A prospective randomized controlled study of experienced intubators. Resuscitation.

[2] Sulser S, Ubmann D, Schlaepfer M (2016). C-MAC videolaryngoscope compared with direct laryngoscopy for rapid sequence intubation in an emergency department. A randomised clinical trial. Eur J Anaesthesiol.

[3] Lascarrou JB, Boisrame-Helms J, Bailly A (2017). Video laryngoscopy vs direct laryngoscopy on successful first-pass orotracheal intubation among ICU patients. A randomized clinical trial. JAMA.

[4] Prekker ME, Driver BE, Trent SA (2023). Video versus direct laryngoscopy for tracheal intubation of critically ill adults. N Engl J Med.

[5] Kongsawaddee T, Kornthatchapong K, Srivilaithon W (2024). Outcome of video laryngoscopy versus direct laryngoscopy for emergency tracheal intubation in emergency department: a propensity score matching analysis. BMC Emerg Med.

[6] Yuan J, Yang P, Yu L, Zhang W, Yu J, Chen Q (2025). Comparison of video laryngoscopy with direct laryngoscopy in critically ill patients: a systematic review and meta-analysis of randomized controlled trials. Eur J Med Res.

[7] Azam S, Khan ZZ, Shahbaz H (2024). Video versus direct laryngoscopy for intubation: updated systematic review and meta-analysis. Cureus.

[8] Park SO, Kim JW, Na JH, Lee KH, Lee KR, Hong DY, Baek KJ (2015). Video laryngoscopy improves the first-attempt success in endotracheal intubation during cardiopulmonary resuscitation among novice physicians. Resuscitation.

[9] Declercq PL, Bubenheim M, Gelinotte S (2016). Usefulness of video-laryngoscopy with the Airway Scope for intubation performance and learning: an experimental manikin controlled study. Ann Intensive Care.

[10] Liu DX, Ye Y, Zhu YH, Li J, He HY, Dong L, Zhu ZQ (2019). Intubation of non-difficult airways using video laryngoscope versus direct laryngoscope: a randomized, parallel-group study. BMC Anesthesiol.

[11] Eismann H, Sieg L, Etti N (2017). Improved success rates using videolaryngoscopy in unexperienced users: a randomized crossover study in airway manikins. Eur J Med Res.

[12] Sugaya A, Naito K, Goto T, Hagiwara Y, Okamoto H, Watase H, Hasegawa K (2023). First-pass success of video laryngoscope compared with direct laryngoscope in Intubations performed by residents in the emergency department. Cureus.

[13] Carlson RV, Boyd KM, Webb DJ (2004). The revision of the Declaration of Helsinki: past, present and future. Br J Clin Pharmacol.

[14] Baek MS, Han M, Huh JW, Lim CM, Koh Y, Hong SB (2018). Video laryngoscopy versus direct laryngoscopy for first-attempt tracheal intubation in the general ward. Ann Intensive Care.

[15] Sakles JC, Mosier JM, Patanwala AE, Arcaris B, Dicken JM (2016). The utility of the C-MAC as a direct laryngoscope for intubation in the emergency department. J Emerg Med.

[16] Chaudery H, Hameed H, Sharif Z, Asinger S, McKechnie A (2024). Comparative efficacy of videolaryngoscopy and direct laryngoscopy in patients living with obesity: a meta-analysis. Cureus.

[17] Trent SA, Kaji AH, Carlson JN, McCormick T, Haukoos JS, Brown CA 3rd (2021). Video laryngoscopy is associated with first-pass success in emergency department intubations for trauma patients: a propensity score matched analysis of the national emergency airway registry. Ann Emerg Med.

[18] Knapp J, Eberle B, Bernhard M, Theiler L, Pietsch U, Albrecht R (2021). Analysis of tracheal intubation in out-of-hospital helicopter emergency medicine recorded by video laryngoscopy. Scand J Trauma Resusc Emerg Med.

[19] Frerk C, Mitchell VS, McNarry AF (2015). Difficult Airway Society 2015 guidelines for management of unanticipated difficult intubation in adults. Br J Anaesth.

[20] Ali TO, El-Boghdadly K (2024). The role of cricoid pressure in rapid sequence induction. Curr Anesthesiol Rep.

[21] Arulkumaran N, Lowe J, Ions R, Mendoza M, Bennett V, Dunser MW (2018). Videolaryngoscopy versus direct laryngoscopy for emergency orotracheal intubation outside the operating room: a systematic review and meta-analysis. Br J Anaesth.

[22] Hansel J, Rogers AM, Lewis SR, Cook TM, Smith AF (2022). Videolaryngoscopy versus direct laryngoscopy for adults undergoing tracheal intubation: a Cochrane systematic review and meta-analysis update. Br J Anaesth.

[23] Noguchi T, Koga K, Shiga Y, Shigematsu A (2003). The gum elastic bougie eases tracheal intubation while applying cricoid pressure compared to a stylet. Can J Anaesth.

